# Forensic entomology

**DOI:** 10.1080/20961790.2017.1403081

**Published:** 2017-12-08

**Authors:** Jens Amendt

**Affiliations:** Institute of Legal Medicine, Goethe-University, Frankfurt, Germany

For many members of the forensic community, insects still have an exotic status. This may be one reason why forensic entomology, the analysis of insect evidence for forensic and legal purposes, has not yet achieved the significance it deserves in forensic sciences. The present special issue may help to change that.

From a scientific point of view, the study of necrophagous insects offers an enormously diverse range of topics. Physiological growth rates, ecological succession, molecular species identification or biochemical profiling – all of these exemplars play a part in the field of forensic entomology and illustrate the wide variety of methods. This is very inspiring from a scientific point of view – since Smith's “Manual of Forensic Entomology” (1986), about 900 original international scientific papers, reviews and case studies as well as various textbooks on forensic entomology have been published. The present special edition also illustrates this thematic diversity with contributions about development, molecular and morphological identification, histology and sampling of evidence.

The exciting fact, from a scientific perspective, that there are still blank spots on the research map to be eradicated, and that the results of some work might only give rise to new questions and studies, but no final answers, is double-edged for a forensic discipline. Forensic Science demands reliability and the fulfilment of specified criteria, illustrated by international standards like the Daubert standard or ISO 17025. In the last 10–15 years, substantial progress has been made in this field of forensic entomology, including the accreditation of the entomological laboratories of the French Institut de Recherche Criminelle de la Gendarmerie Nationale and the laboratory of the Belgium Institut National de Criminalistique et Criminologie, two of Europe's leading forensic institutes, the publication of standards for the sampling and evaluation of entomological traces by the European Association for Forensic Entomology and the certification of entomological experts by the American Board of Forensic Entomology.

We may now perhaps reach a state where it is necessary to weigh how many guidelines and standards are needed to force forensic entomology into a not too narrow corset, which even might hamper the further development of our discipline. A reliable chain of custody and associated steps and processes for sure improve the quality outcome of an entomological expertise. But in every report, there will be not just an evidence of fact, but also an evidence of opinion, as entomological expertise is often based not only on the expert's knowledge, but also on his or her experience. It has to be acknowledged by the judges that each case has unique features and maybe not just one answer.

Nevertheless one of the major challenges in the nearest future of forensic entomology will be, that, despite these unique features and the different experiences of the reviewers, the same result should be achieved in the final report. The introduction of inter-laboratory tests and the analysis of mock samples, such as the ones carried out by the European Association for Forensic Entomology since several years, will help a lot here. I hope that Forensic Sciences Research can be a platform for spreading the word and supporting the development of other natural history approaches like palynology or nematodology. In the future, classical forensic scientists like, for example, forensic pathologists and natural history academics must have to work together even more closely – this is the only way to ask the right questions and provide answers with an appropriate forensic and scientific expertise. Such a tight connection also would help to bridge the gap between basic research and applied science, and to avoid oversized expectations but instead give realistic answers.

